# Peroxiredoxin 6 promotes upregulation of the prion protein (PrP) in neuronal cells of prion-infected mice

**DOI:** 10.1186/1478-811X-10-38

**Published:** 2012-12-04

**Authors:** Wibke Wagner, Andreas Reuter, Petra Hüller, Johannes Löwer, Silja Wessler

**Affiliations:** 1 Paul Ehrlich Institute, Paul Ehrlich-Straße 51-59, Langen, D-63225, Germany; 2 Division of Microbiology, University of Salzburg, Billrothstrasse 11, Salzburg, A-5020, Austria; 3 Present address: Department of Biology, Division of Neurosensory systems, Technical University of Darmstadt, Schnittspahnstraße 10, Darmstadt, D-64287, Germany; 4 Present address: Chiltern International GmbH, Norsk-Data-Strasse 1, Bad Homburg v.d.H, D-61352, Germany

**Keywords:** Peroxiredoxin 6, Prion protein, Scrapie

## Abstract

**Background:**

It has been widely established that the conversion of the cellular prion protein (PrP^C^) into its abnormal isoform (PrP^Sc^) is responsible for the development of transmissible spongiform encephalopathies (TSEs). However, the knowledge of the detailed molecular mechanisms and direct functional consequences within the cell is rare. In this study, we aimed at the identification of deregulated proteins which might be involved in prion pathogenesis.

**Findings:**

Apolipoprotein E and peroxiredoxin 6 (PRDX6) were identified as upregulated proteins in brains of scrapie-infected mice and cultured neuronal cell lines. Downregulation of PrP gene expression using specific siRNA did not result in a decrease of PRDX6 amounts. Interestingly, selective siRNA targeting PRDX6 or overexpression of PRDX6 controlled PrP^C^ and PrP^Sc^ protein amounts in neuronal cells.

**Conclusions:**

Besides its possible function as a novel marker protein in the diagnosis of TSEs, PDRX6 represents an attractive target molecule in putative pharmacological intervention strategies in the future.

## Findings

Transmissible spongiform encephalopathies (TSEs) are fatal neurodegenerative disorders, which include scrapie in sheep, bovine spongiform encephalopathy (BSE) in cattle, and Creutzfeldt-Jacob disease (CJD) in humans [1]. The molecular hallmark of these disorders is a structural conversion in folding of the normal cellular prion protein (PrP^C^) into a disease-associated, protease-resistant isoform (PrP^Sc^) [2]. Neuropathological characteristics of these diseases include neuronal loss, vacuolar degeneration, astrogliosis and amyloid plaque formation caused by accumulation of PrP^Sc^[3]. However, the mechanism whereby PrP^C^ → PrP^Sc^ conversion triggers cellular neurotoxicity and neurodegeneration is not well understood.

PrP^C^ is a multifunctional plasma membrane glycosylphosphatidylinositol (GPI)-anchored protein on a wide range of different cell types where it is involved in adhesion, signal transduction, differentiation, survival or stress protection [4-6]. Obviously, neurodegenerative disorders interconnect several cellular signal transduction pathways to cause oxidative stress in the brain, including increased oxidative damage, impaired mitochondrial function, defects of the proteasome system, the presence of aggregated proteins, and many more [7]. There are a number of cellular antioxidant defenses to convert reactive oxygen species (ROS) into unreactive compounds, e.g. superoxide dismutase (SOD) and the peroxiredoxin (PRDX) family. Proteins of the PRDX family are widely expressed and exhibit crucial protective functions in neurological disorders such as Parkinson's and Alzheimer's diseases [8]. Accordingly, upregulation of PRDX protein was observed in the brain of patients with Parkinson and Alzheimer’s disease, and also during experimental prion infection in mice [9-11]. The PRDX family of mammals comprises six isoforms (PRDX1-6), which are classified into the three subgroups typical 2-Cys PRDX (PRDX1–4), atypical 2-Cys PRDX (PRDX5) and 1-Cys PRDX (PRDX6) [12]. Peroxiredoxin 6 is the only 1-Cys member and exhibits bifunctional activities as a glutathione (GSH) peroxidase and calcium-independent phospholipase A_2_ (PLA_2_) [13], which might contribute to neurological disorders. In experimental *in vivo* models for neurological disorders such as Huntington^′^s disease and scrapie, PRDX1 was slightly enhanced [11]. However, the function of distinct PRDX isoforms in prion diseases has not been investigated.

### Upregulation of PRDX6 in scrapie-infected brains

**Figure 1:**
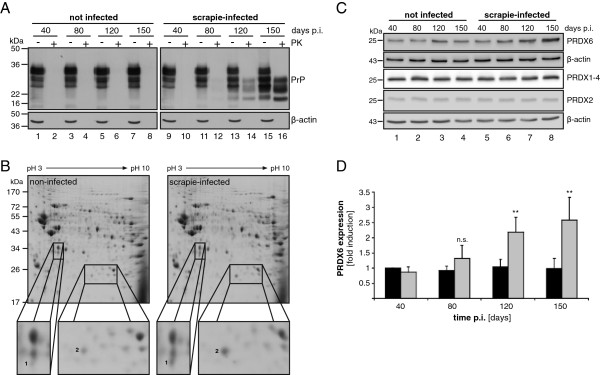
**Upregulation of PRDX6 in scrapie-infected mice.** (**A**) C57Bl/6 mice were inoculated intracerebrally with PBS (not infected, lanes 1–8) or 10% brain homogenate of the prion strain 139A (scrapie-infected, lanes 9–16). 40, 80, 120 and 150 days post infection (p.i.) brain homogenates were prepared and incubated with 20 μg/ml proteinase K (+, PK) or left untreated (−) prior to Western blotting. PrP was detected using the 8H4 monoclonal antibody showing the typical migration pattern of PrP and PK-resistant PrP^res^. β-actin was shown as a loading control. (**B**) 150 μg protein of brain homogenates were separated by two-dimensional gel electrophoresis followed by Coomassie blue staining. Enlarged sections indicate differentially expressed proteins (spots 1 and 2) observed in four tested homogenates. (**C**) 50 μg protein of brain homogenates were separated by SDS-PAGE followed by Western blot. PRDX6, PRDX1-4, PRDX2 and β-actin were detected using specific antibodies. (**D**) Quantification of protein expression was performed using four independent Western blots detecting PRDX6 (grey bars) and β-actin (black bars) in brain homogenates of four different mice, respectively. PRDX6 expression was by normalized to β-actin (**, *p* < 0.01, n.s., non-specific)

For a better understanding of the proteomic alterations during *in vivo* prion pathogenesis, C57Bl/6 mice were intracerebrally inoculated with a 10% brain homogenate containing the prion strain 139A (Additional file 1). Mice were sacrificed after 40, 80, 120 and 150 days, and brain homogenates were prepared. To confirm prion infection, PrP expression was analyzed by Western blot (Figure 1A). PrP^C^ was detected in non-infected brain homogenates in equal amounts 40, 80, 120 and 150 days post infection (p.i.) (Figure 1A, lanes 1, 3, 5, 7), while total PrP of scrapie-infected brain homogenates slightly increased during infection (Figure 1A, lanes 9, 11, 13, 15), caused by the accumulation of PrP^Sc^ as demonstrated by proteinase K (PK)-digestion (Figure 1A, lanes 10, 12, 14, 16).

To identify differentially regulated proteins in scrapie-infected mice, equal protein amounts of brain homogenates were separated by two-dimensional gel electrophoresis followed by Coomassie blue staining (Figure 1B). Only two up-regulated protein spots were reproducibly detected in four individual mice infected by scrapie, which were then identified by mass spectrometry (Table 1). Apolipoprotein E was found in three out of four investigated samples, while peroxiredoxin 6 (PRDX6) was identified with an overall sequence coverage of 71.4% in all four tested samples (Table 1). Upregulation of apolipoprotein E has already been described in activated astrocytes in Alzheimer′s and prion disease [14] and is considered as one of the strongest genetic risk factors in Alzheimer disease [15]. Contrastingly, only marginal information is available on the expression of peroxiredoxins in prion disease. Peroxiredoxin protein was preferentially upregulated in astrocytes of prion-infected mouse brains [10], but it remained unknown whether all PRDX family members or a single isoform accumulated. Furthermore, PRDX6 protein expression in astrocytes has already been associated with Alzheimer disease where it might function as an antioxidant enzyme [9] suggesting that PRDX6 might be involved in neurological diseases.

**Table 1:** Identification of apolipoprotein E and peroxiredoxin 6 by mass spectrometry

**Spot**	**Mouse**	**Acc.nr.**	**Protein name**	**Score**	**Peptide**	**Sequence coverage (%)**
1	1	P08226	Apolipoprotein E OS Mus musculus GN Apoe PE 1 SV 2	470.7	5	19.3
1	3	P08226	Apolipoprotein E OS Mus musculus GN Apoe PE 1 SV 2	220.7	3	13.5
1	4	P08226	Apolipoprotein E OS Mus musculus GN Apoe PE 1 SV 2	215.3	4	16.4
2	1	O08709	Peroxiredoxin 6 OS Mus musculus GN Prd × 6 PE 1 SV 3	1023.0	10	59.4
2	2	O08709	Peroxiredoxin 6 OS Mus musculus GN Prd × 6 PE 1 SV 3	1176.2	11	69.6
2	3	O08709	Peroxiredoxin 6 OS Mus musculus GN Prd × 6 PE 1 SV 3	1737.0	11	59.8
2	4	O08709	Peroxiredoxin 6 OS Mus musculus GN Prd × 6 PE 1 SV 3	779.9	7	33.5

To investigate this in more detail, we followed PRDX6 expression during scrapie infection in mice and compared it with PRDX1-4 amounts by Western blot analyses (Figure 1C). In contrast to non-infected mice (Figure 1C, lane 1–4), the level of PRDX6 steadily increased within 150 days post infection (Figure 1C, lane 5–8). This appears to be highly specific, since amounts of PRDX1-4 or PRDX2 did not change during infection (Figure 1C, lane 5–8). Although we cannot exclude the possibility that PRDX1, 3 or 4 of the 2-Cys PRDX subgroup were slightly regulated, it is obvious that PRDX6 was strongly affected in scrapie-infected mice brains. Quantification of PRDX6 protein expression in brains of four individual mice at each time point after infection indicated that the observed effect was statistically significant and reproducibly observed in scrapie-infected mice (Figure 1D).

### Expression of peroxiredoxin 6 in PrP deficient and PrP^C^ expressing neuronal precursor cells

**Figure 2:**
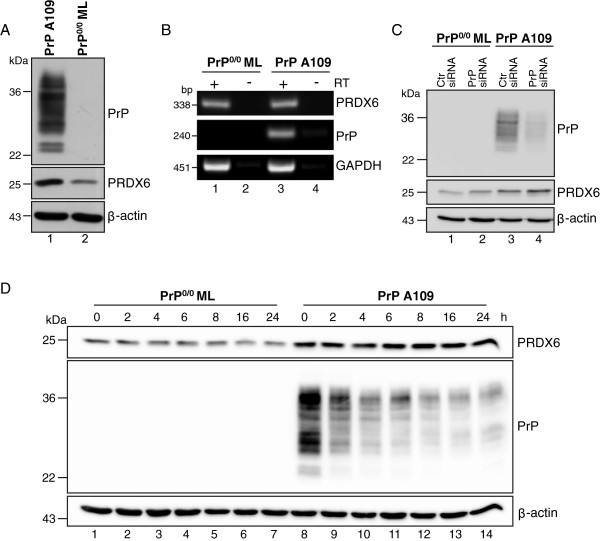
**PRDX6 upregulation is not strictly dependent on PrP expression.** (**A**) Lysates of PrP-deficient (PrP^0/0^) and PrP^C^-expressing cells (PrP) were analyzed for PrP^C^, PRDX6 and β-actin. (**B**) Semi-quantitative PCR was performed with specific primers for murine *prdx6*, *prnp* and *gapdh* with (+) or without (−) reverse transcriptase (RT). (**C**) PrP^0/0^ and PrP cells were transfected with control siRNA (Ctr. siRNA) or siRNAs specific for murine PrP (PrP siRNA). 72 h after transfection cell lysates were prepared and Western blot analysis using the specific anti-PrP antibody SAF32 and anti-PRDX6 antibody were performed. β-actin was shown as a loading control. (**D**) Cells were incubated with 5 μg/ml cycloheximide and lysed after indicated time points. Equal amounts of cell lysates were separated by SDS-PAGE and detection of PRDX6, PrP and β-actin was carried out using specific antibodies

To investigate PRDX6 expression in more detail, endogenous PRDX6 expression in PrP-deficient and PrP^C^-expressing cells was analyzed. The immortalized neuronal precursor cell line PrP^0/0^ ML derived from PrP^0/0^ ZrchI mice was stably transfected with wild type murine PrP (PrP A109) [16]. Western blots of these cell lines were performed for detection of PrP and PRDX6. As expected, PrP A109 cells expressed PrP^C^, whereas the PrP^0/0^ ML cells did not (Figure 2A, upper panel). Corresponding to *in vivo* studies, PRDX6 expression was increased in PrP A109 cells (Figure 2A, middle panel) while detection of β-actin indicated equal protein loading (Figure 2A, lower panel). Analyzing mRNA synthesis of *prdx6* and *prnp*, no significant alterations were observed indicating that PRDX6 protein expression was not transcriptionally dependent on PrP (Figure 2B). To clarify whether PrP protein expression led to PRDX6 accumulation, PrP was downregulated using specific siRNAs and the amount of PRDX6 protein was analyzed by Western blotting. Interestingly, successful downregulation of PrP (Figure 2C, lane 4) did not result in a detectable decrease in PRDX6 protein amount (Figure 2C, lane 4). This finding was further supported by the inhibition of protein translation using cycloheximide. Twenty-four hours incubation of the cells with cycloheximide led to a drastic decrease in PrP expression, while leaving PRDX6 protein amounts unaffected (Figure 2D, lanes 8–14). Although downregulation of PrP was not complete, these data imply that enhanced PrP expression does not induce PRDX6 expression and that PRXD6 is highly stable in neuronal cells.

### PRDX6 induces upregulation of PrP in neuronal cells

**Figure 3:**
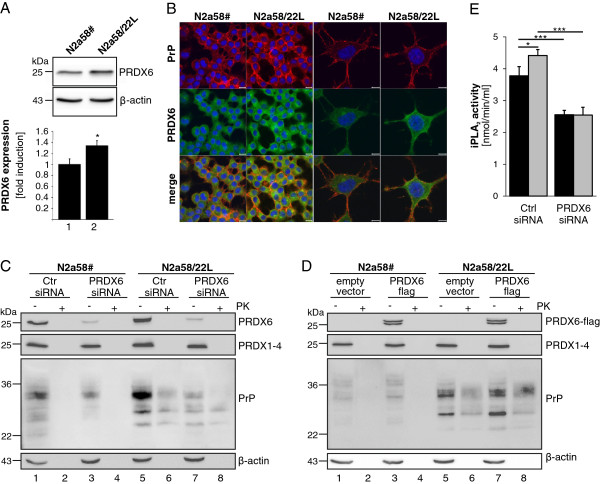
**PRDX6 induces PrP upregulation.** (**A**) Detection of endogenous PRDX6 in uninfected N2a58# and scrapie-infected N2a58/22L cells was performed by Western blot using specific anti-PRDX6 and anti-β-actin antibodies (upper panel). Quantification of PRDX6 expression was carried out of four independent experiments (lower panel; *, *p* < 0.05). (**B**) PrP (red) and PRDX6 (green) in uninfected N2a58# and scrapie-infected N2a58/22L cells were detected by laser scanning microscopy using PrP-specific 6H4 and PRDX6 antibodies. Nuclei (blue) were stained by DAPI. Scale bar, 10 μm (left panel); scale bar, 5 μm. (**C**) Downregulation of PRDX6 was performed by reverse transfection of a control siRNA (Ctr. siRNA) or a combination of two siRNAs specific for PRDX6 (PRDX6 siRNA). 48 h after transfection cells were lysed and either incubated with 20 μg/ml PK (+) or left untreated (−). Western blot was performed using specific antibodies against PRDX6, PRDX1-4, PrP (8H4) and anti-β-actin antibodies. (**D**) Cells were transfected with an empty vector control or a PRDX6 expression plasmid (PRDX6-flag) and lysed 24 h after transfection. Equal amounts of cell lysates were treated with PK (+) or left untreated (−) following SDS-PAGE and immunoblotting using anti-flag, anti-PRDX6, anti-PRDX1-4, anti-PrP (8H4) and anti-β-actin antibodies. (**E**) The activity of iPLA_2_ was analyzed in N2a58# (black bars) and scrapie-infected N2a58/22L cells (grey bars), which were either treated with siRNA targeting PRDX6 or control siRNA (Ctrl) (*, *p* < 0.05; ***, *p* < 0.001)

Next, we aimed at the investigation of PRDX6 expression in scrapie-infected neuronal cells. Uninfected PrP^C^ expressing N2a58# cells were compared to scrapie-infected N2a58/22L cells. Correspondingly to scrapie-infected mice, an upregulation of PRDX6 was observed in scrapie-infected N2a58/22L cells as shown by Western blot analysis (Figure 3A, upper panel). Signal intensities from four independent experiments were quantified and expressed as fold PRXD6 expression normalized to β-actin expression (Figure 3A, lower panel). Increased PRXD6 protein amounts were also detected by immunofluorescence analyses underlining enhanced protein occurrence in the cytoplasm, while PrP was mainly localized at the plasma membrane (Figure 3B).

Since PRDX6 was upregulated in PrP^C^-expressing and PrP^Sc^-infected cells, but not in cells in which PrP^C^ expression was downregulated, we tested the hypothesis if PRDX6 is involved in PrP upregulation in turn. In N2a58# and N2a58/22L cells PRDX6 was successfully downregulated using PRDX6 specific siRNA that did not affect PRDX1-4 expression. Interestingly, PrP^C^ in N2a58# cells was slightly decreased and PK-resistant PrP^Sc^ was strongly reduced in N2a58/22L upon siRNA treatment to inhibit PRXD6 expression (Figure 3C). These data led to the suggestion that there is a functional connection between PrP and PRDX6 expression. Therefore, flag-tagged PRDX6 was overexpressed in N2a58# and N2a58/22L cells and the amount of PrP^C^ and PrP^Sc^ was examined. Overexpression of PRDX6-flag had no influence on expression of PRDX1-4 (Figure 3D). However, PRDX6-flag resulted in a slightly increased amount of PrP^C^ in uninfected N2a58# (Figure 3D, lanes 1–4) and subsequently to an obvious accumulation of PK-sensitive PrP^C^ and PK-resistant PrP^Sc^ in infected N2a58/22L (Figure 3D, lanes 5–8). PRDX6 exhibits a calcium-independent phospholipase A2 (iPLA_2_) activity [17]. In N2a58/22L cells, iPLA_2_ activity was significantly increased compared to N2a58# cells. Importantly the difference was completely diminished after down-regulation of PRDX6 in both cell lines (Figure 3E). In conclusion, these results suggest that the expression level and activity of PRDX6 might be involved in the control of the level of PrP^C^ and subsequently PrP^Sc^ conversion.

In this study, enhanced amounts of PRDX6 was selectively identified in brains of prion-infected mice and neuronal cell lines concomitant with an increased amount of PrP^C^ and consequently of PrP^Sc^. This interaction appears very complex, since PrP^C^ expression in PrP knock-out cells has also been observed to increase the amount of PRDX6 in turn, but downregulation of PrP did not alter PRDX6 appearance. This effect could be explained by the observation that the PRDX6 protein was more stable than PrP. Hence, the molecular basis for this phenomenon remains unknown, but might indicate a complex “tandem”-regulation of PRDX6 and PrP.

PRDX6 is a moonlighting protein containing peroxidase and PLA_2_ activities [18]. Specific pharmacological inhibitors for cellular studies are not available, but it is tempting to speculate whether PRDX6 activities are involved in PrP regulation. In fact, data are accumulating that PLA_2_ contributes to prion diseases. Functionally, PLA_2_ is an important promoter of phospholipid metabolism and cleaves membrane phospholipids to produce arachidonic acid and lysophopholipids as major products [19]. Under normal conditions, arachidonic acid is either re-incorporated into phospholipids, converted to inflammatory mediators in the brain or modulates neuronal functions [20]. It has been demonstrated that PrP^Sc^ and the neurotoxic PrP106-126 prion peptide stimulated the N-methyl-D-aspartate (NMDA) receptor [21], which is accompanied by the release of arachidonic acid, suggesting an involvement of PLA_2_ in prion pathogenesis [22]. This has been supported by neuronal cell culture studies showing that PLA_2_ is activated by glycosylphosphatidylinositols (GPIs) isolated from PrP^C^ and PrP^Sc^[23]. Interestingly, treatment of CJD using the non-specific PLA_2_ inhibitor quinacrine resulted in an inhibition of PrP^Sc^ formation [24] and reduced toxicity of PrP106-126 [25]. Together with our study, those data point to PRDX6 activities as new important players in the pathogenesis of prion diseases.

## Abbreviations

PRDX6: Peroxiredoxin 6; PrP: Prion protein.

## Competing interests

The authors declare that they have no competing interests.

## Authors’ contributions

WW: performed the experiments and wrote the manuscript. AR: conducted and interpreted mass spectrometry analysis. PH: performed the animal experiments. JL: participated in the design of the study and the interpretation of the results. SW: conceived of the study, and participated in design and coordination and wrote the manuscript. All authors read and approved the final manuscript.

## Supplementary Material

Additional file 1 Wagner et al., Cell Communication and Signaling. Click here for file
