# Supplementary material for: Peroxiredoxin 6 promotes upregulation of the prion protein (PrP) in neuronal cells of prion-infected mice
Source: Cell Commun Signal. 2012 Dec 4;10:38. doi: 10.1186/1478-811X-10-38 (PMC3519792; doi:10.1186/1478-811X-10-38)
Supplement: Additional file 1 — Wagner et al., Cell Communication and Signaling. [file 1478-811X-10-38-S1.docx]

**Additional File 1**

**Wagner et al., Cell Communication and Signaling**

**Material and Methods**

*Murine brain samples and cell lines*

C57Bl/6 mice were intracerebrally inoculated with 20 µl PBS or 10% brain homogenate of scrapie strain 139A (TSE Resource Center) and sacrificed 40, 80, 120 and 150 days post inoculation. Immortalized neuronal precursor cell lines PrP^0/0^ ML and PrP A109 have been described previously [[1](#_ENREF_1)] and were cultured in DMEM containing 10% FCS and 4 mM L-glutamine at 33°C. N2a58/22L cells have been described previously and were kindly provided by Prof. Schätzl (LMU, Munich) [[2](#_ENREF_2)]. N2a58/22L cells were cultured in DMEM containing 10% FCS and 4 mM L-glutamine at 37°C. Treatment with 5 μg/ml pentosan polysulfate (Cartrophen Vet) for two passages resulted in a stable rescued cell line for more than 15 passages (N2a58# cells). Where indicated, cells were incubated with 5 µg/ml cycloheximide to inhibit protein translation. Brain homogenates and cell lysates were prepared in lysis buffer containing 150 mM NaCl, 0.5% Triton X-100, 0.5% DOC, 50 mM Tris pH 7.5, 1 mM Na-vanadate, 1 mM Na-molybdate, 20 mM NaF, 10 mM NaPP, 20 mM β-glycerophosphate, 1× protease inhibitor cocktail (Roche). For digestion with proteinase K (PK) 80 μg protein were treated with 20 μg/ml PK for 30 min at 37°C. PK digestion was stopped by addition of laemmli sample buffer and protein denaturation at 95°C for 7 min.

*Cloning of PRDX6, transfection of plasmids and siRNA*

PRDX6 was amplified from N2a58# cells using specific primers (5’-ATC GAA TTC ATG CCC GGA GGG-3’ and 5’-GAT AAG CTT AGG CTG GGG TGT-3’) and cloned into pCMV-3tag-3A vector (Stratagene) to create a Prdx6-flag fusion protein. Cells were transfected with PRDX6-flag using Lipofectamine 2000 in OptiMEM (Invitrogen) following the manufacturer’s instructions. A combination of two PRDX6-specific siRNAs (5‘-GGC UCU CUU UGA AAC GAA Utt-3‘ and 5‘-GUU GAU UGC UCU UUC AAU Att-3‘, Santa Cruz) were reverse transfected using Dharmafect (Dharmacon).

*SDS-PAGE and Western Blot*

Proteins were separated by 12% SDS-PAGE and transferred to polyvinylidene difluoride membranes (PVDF, Millipore) by semidry blotting. PrP was detected using the PrP-specific mouse mAb 8H4 (Alicon AG) and SAF32 (Cayman Chemicals). For validation of peroxiredoxins anti-PRDX1-4 (sc-33574, Santa Cruz Biotechnologies), anti-PRDX2 (ab71533, Abcam), and anti-PRDX6 antibodies (ab16947, Abcam) were used. Prdx1-4 (sc-33574) was a rabbit polyclonal antibody raised against amino acids 1-198 of PRX I-IV. The anti-PRDX2 antibody (ab71533) was produced using recombinant mouse protein purified from *E. coli*. The anti-PRDX6 antibody (ab16947) was a mouse monoclonal antibody [4A3] produced using recombinant full length protein. Antibodies recognizing β-actin were obtained from Sigma-Aldrich. Where indicated, Western blots were quantified using the Fusion FX-7 image analyzer and Bio-1D analysis software (Vilber Lourmat). All experiments were repeated at least three times.

*Immunofluorescence*

For immunofluorescence staining, cells were fixed with 4% paraformaldehyde in PBS, permeabilized with 0.2% Triton X-100 in PBS and blocked with 1% bovine serum albumin. Proteins were stained with anti-PrP 6H4 (Prionics Deutschland GmbH) and anti-PRDX6 antibodies (Abcam). For nuclei staining, 4,6-diamidino-2-phenylindole (Roche) was used as recommended by the manufacturer. Samples were analyzed by confocal laser scanning microscopy using a Zeiss LSM 510 Meta confocal microscope.

*2-Dimensional gel electrophoresis and mass spectrometry*

For 2D electrophoresis 150 μg protein of cell lysates were purified by trichloroacetic acid precipitation and re-suspended in DeStreak Rehydration Solution (Amersham Biosciences) containing 0.5% Bio-Lyte pH 3-10 (Bio-Rad Laboratories GmbH). The isoelectric focusing was run on IPG strips with a non-linear pH range of 3-10 and a length of 7 cm (Bio-Rad) using the ZOOM® IPG Runner™ system from Invitrogen. After focusing strips were equilibrated in 50 mM Tris, 1 mM urea, 30% glycerine, 2% SDS, 1% DTT for 25 min and in 50 mM Tris, 1 mM urea, 30% glycerine, 2% SDS, 5% iodacetamid for 25 min. Strips were then separated in 10% SDS-PAGE gels in the second dimension and analyzed by Coomassie Blue staining. Proteins of interest were excised and subjected to tryptic digestion as described elsewhere [[3](#_ENREF_3)]. Differing from this protocol, peptides were eluted with 25 mM NH_4_HCO_3_; 10% aceto nitrile (ACN) and the digestion was stopped by adding 5% formic acid. The peptides were separated with a nano-Acquity UPLC (Waters, Milford, US) using a 5 µm symmetry 180 µm x 20 mm c18 pre-column and a 1.7 µm BEH 130 100 µm x 100 mm c18 separation column at a flow rate of 500 nl applying a gradient of 30 minutes (3% ACN to 40% ACN). The UPLC was coupled to a nano-ESI synapt mass spectrometer (Waters) operated in V mode, acquiring MSE data and applying standard parameters. Data analysis was performed with protein lynx global server version 2.3 (waters) by searching the uniprot database restricted to reviewed entries. Protein hits were accepted at a false positive rate of less than 4%. Peptide mass accuracy was 5ppm or better.

*RNA-Isolation and RT-PCR*

Total RNA was isolated using the RNeasy mini kit (Qiagen GmbH) following DNaseI treatment (Invitrogen). First-strand cDNA was synthesized from 1 µg of total RNA using SuperScript III First-Strand Synthesis System with oligo(dT) primer (Invitrogen) following the manufacturer’s instructions.

2µl cDNA was amplified by PCR for *prdx6* (fw: 5‘-ATG CCC GGA GGG TTG CTT CT-3‘, rev: 5‘-AAG GAT GGC AAG GTC CCT GC-3‘), *prnp* (fw: 5‘-AAA AAG CGG CCA AAG CCT-3‘, rev: 5‘-TGC TGG GCT TGT TCC ACT-3‘) and *gapdh* (fw: 5‘-ACC ACA GTC CAT GCC ATC AC-3‘, rev: 5‘-TCC ACC ACC CTG TTG CTG TA-3‘). In brief, cDNA were subjected to PCR in a 25 µl reaction solution, containing 2.5 µl 10x PCR buffer with 1.5 mM MgCl_2_, 300 µM dNTPs, 0.5 µM of both primers (forward and reverse) and 1 U AmpliTaq® DNA Polymerase (Applied Biosystems).

iPLA_2_ Activity Assay

iPLA_2_ activities were measured according to the manufacturer's recommendations (Cayman Chemicals, Biomol GmbH, Hamburg) with some minor modifications. Cells were scraped into 50 mM Hepes, pH 7.4, containing 1 mM EDTA, sonicated and centrifuged at 10,000 × g for 15 min at 4°C. Forty μg protein of the supernatant in a total volume of 10 μl were added to microplate wells containing 5 μl of calcium-free iPLA_2_ activity assay buffer (10 mM Hepes, 300 mM NaCl, 8 mM Triton X-100, 60% glycerine, 2 mg/ml BSA, 4 mM EGTA). The reaction was initiated by addition of 200 μl of 1.5 mM arachidonoyl thiophosphatidylcholine and was incubated at room temperature for 60 min. The reaction was terminated by addition of 10 μl of 5,5′-dithio-bis(2-nitrobenzoic acid), and the absorbance was measured at 405 nm in a microplate reader (Genios plus, Tecan). The iPLA_2_ activity was calculated according to the manufacturer's instructions. Evaluation was performed by means of a mixed linear model with fixed factor treatment group and random effect assay (for each group, data from 2 assays with each 3 replicates were available). P-values and 95% Confidence Intervals were adjusted according Bonferroni for multiple comparisons. The statistical analysis was performed with SAS®/STAT software, version 9.3, SAS System for Windows. For statistical significant results the following convention was used: * – p-value < 0.05, ** – p-value < 0.01 and *** – p-value < 0.001.

**References**

1. Barenco MG, Valori CF, Roncoroni C, Loewer J, Montrasio F, Rossi D: **Deletion of the amino-terminal domain of the prion protein does not impair prion protein-dependent neuronal differentiation and neuritogenesis.** *J Neurosci Res* 2009, **87:**806-819.

2. Wagner W, Ajuh P, Lower J, Wessler S: **Quantitative phosphoproteomic analysis of prion-infected neuronal cells.** *Cell Commun Signal* 2010, **8:**28.

3. Albrecht M, Alessandri S, Conti A, Reuter A, Lauer I, Vieths S, Reese G: **High level expression, purification and physico- and immunochemical characterisation of recombinant Pen a 1: a major allergen of shrimp.** *Mol Nutr Food Res* 2008, **52 Suppl 2:**S186-195.
